# The Diabits App for Smartphone-Assisted Predictive Monitoring of Glycemia in Patients With Diabetes: Retrospective Observational Study

**DOI:** 10.2196/18660

**Published:** 2020-09-22

**Authors:** Stan Kriventsov, Alexander Lindsey, Amir Hayeri

**Affiliations:** 1 Bio Conscious Technologies Inc Vancouver, BC Canada

**Keywords:** blood glucose predictions, type 1 diabetes, artificial intelligence, machine learning, digital health, mobile phone

## Abstract

**Background:**

Diabetes mellitus, which causes dysregulation of blood glucose in humans, is a major public health challenge. Patients with diabetes must monitor their glycemic levels to keep them in a healthy range. This task is made easier by using continuous glucose monitoring (CGM) devices and relaying their output to smartphone apps, thus providing users with real-time information on their glycemic fluctuations and possibly predicting future trends.

**Objective:**

This study aims to discuss various challenges of predictive monitoring of glycemia and examines the accuracy and blood glucose control effects of Diabits, a smartphone app that helps patients with diabetes monitor and manage their blood glucose levels in real time.

**Methods:**

Using data from CGM devices and user input, Diabits applies machine learning techniques to create personalized patient models and predict blood glucose fluctuations up to 60 min in advance. These predictions give patients an opportunity to take pre-emptive action to maintain their blood glucose values within the reference range. In this retrospective observational cohort study, the predictive accuracy of Diabits and the correlation between daily use of the app and blood glucose control metrics were examined based on real app users’ data. Moreover, the accuracy of predictions on the 2018 Ohio T1DM (type 1 diabetes mellitus) data set was calculated and compared against other published results.

**Results:**

On the basis of more than 6.8 million data points, 30-min Diabits predictions evaluated using Parkes Error Grid were found to be 86.89% (5,963,930/6,864,130) clinically accurate (zone A) and 99.56% (6,833,625/6,864,130) clinically acceptable (zones A and B), whereas 60-min predictions were 70.56% (4,843,605/6,864,130) clinically accurate and 97.49% (6,692,165/6,864,130) clinically acceptable. By analyzing daily use statistics and CGM data for the 280 most long-standing users of Diabits, it was established that under free-living conditions, many common blood glucose control metrics improved with increased frequency of app use. For instance, the average blood glucose for the days these users did not interact with the app was 154.0 (SD 47.2) mg/dL, with 67.52% of the time spent in the healthy 70 to 180 mg/dL range. For days with 10 or more Diabits sessions, the average blood glucose decreased to 141.6 (SD 42.0) mg/dL (*P*<.001), whereas the time in euglycemic range increased to 74.28% (*P*<.001). On the Ohio T1DM data set of 6 patients with type 1 diabetes, 30-min predictions of the base Diabits model had an average root mean square error of 18.68 (SD 2.19) mg/dL, which is an improvement over the published state-of-the-art results for this data set.

**Conclusions:**

Diabits accurately predicts future glycemic fluctuations, potentially making it easier for patients with diabetes to maintain their blood glucose in the reference range. Furthermore, an improvement in glucose control was observed on days with more frequent Diabits use.

## Introduction

### Background

Diabetes mellitus is one of the biggest public health challenges of our days. Globally, the number of adults living with the disease has risen from 108 to 422 million between 1980 and 2014, constituting about 8.5% of the worldwide adult population [1]. The complications of diabetes caused by increased blood glucose levels (hyperglycemia) include both macrovascular (ischemic heart disease, cerebrovascular disease, peripheral vascular disease leading to lower extremity amputations) and microvascular (eg, diabetic retinopathy and nephropathy) diseases [2].

In healthy adults, the pancreas maintains blood glucose levels between approximately 70 mg/dL and 180 mg/dL [3] (mostly at the lower end of this range, except for short postprandial increases) by balancing the levels of insulin and glucagon in the bloodstream.

Owing to impaired pancreatic function and/or reduced insulin sensitivity, patients with diabetes face the challenge of maintaining their blood glucose levels within the reference range via exogenous insulin administration, medications, and lifestyle modifications (eg, changes in diet, exercise, sleep patterns). These patients, especially those with type 1 diabetes (whose pancreas produces no insulin at all), must constantly monitor their glycemic state and use exogenous insulin to keep their blood glucose from increasing beyond the healthy range into hyperglycemia, while avoiding out-of-range low (hypoglycemic) values, which can potentially lead to seizures, coma, and even death [4].

The task of blood glucose monitoring, traditionally performed using capillary blood sampling, has been made easier in recent years with the introduction of continuous glucose monitoring (CGM) devices [5], which measure glucose levels at a set frequency, typically every 5 min, via interstitial fluid. Currently, CGM devices are capable of providing an accurate picture of recent and current blood glucose levels and alerting the users of hypo- or hyperglycemic events. Some of the existing devices have incorporated simple autoregression algorithms to predict impending blood glucose fluctuations (usually no more than 15-20 min ahead of time) and issue a notification if a hypo- or hyperglycemic event is expected. However, we believe that the functionality of CGM devices can be significantly extended with additional tools to improve their utility and, consequently, the quality of life of their users.

### Current Research on Blood Glucose Predictions

There are two common reasons for making blood glucose predictions. The first is to be able to manage blood glucose levels automatically via a closed-loop feedback system for a continuous insulin pump [6,7]. The second, which is the way in which predictions are used in Diabits, the diabetes management app whose predictive approach and accuracy are reviewed in this publication, is to give the results back to the patient so that their insulin and food intake and other behaviors can be corrected to avoid possible hypo- or hyperglycemia.

Owing to the potential benefits of anticipating blood glucose changes ahead of time, there have been many studies (eg, [8-41]) dedicated to developing models capable of short-term (usually in the range of 15-120 min into the future) glycemic predictions. These studies generally fall into 2 categories: (1) physiological approaches [8-12], wherein researchers try to model the metabolic processes within the patient’s body using general knowledge of human physiology, and (2) data-driven models [13-41], which mostly rely on statistical and machine learning techniques applied to the existing CGM data and other available information (eg, meals, exogenous insulin, sleep, and physical activity) to derive standard patterns of blood glucose behavior, which are then used to predict future glycemic events.

The challenge of using physiological predictive models lies in the fact that to be accurate, these models require a more detailed description of the current state of the patient’s body than can normally be achieved, and even in the presence of such data (eg, in a clinical setting), the performance of physiological models is limited because of the inherent complexity of the human glucose-insulin dynamics, which makes identification of model parameters a difficult task. Therefore, data-driven models (or hybrid models that combine statistical methods with physiological insights) are more viable in practice for short-term blood glucose predictions, as evidenced by most studies cited above.

The data-driven models reported in the literature use a variety of traditional signal processing [14-23] and machine learning [24-41] methods for making blood glucose predictions. These models normally use recent CGM measurements as the primary predictive input.

Among the methods that are not based on machine learning techniques are those using autoregressive methods [14-18], Kalman filters [19-21], and impulse response techniques [22,23] to extrapolate the existing CGM behavior into the near future. Machine learning methods include neural networks [24-36], support vector machines (SVMs) [37,38], decision trees [39,40], grammatical evolution [41], and other approaches. These methods use supervised learning techniques in which the models of blood glucose behavior created on the basis of past measurements are used to anticipate future changes.

### Evaluation of Prediction Accuracy

The accuracy of short-term blood glucose predictions reported in different studies cannot be easily compared, partly because there exists a great variety of metrics that are used by researchers to evaluate predictive performance, such as the root mean square error (RMSE), mean absolute relative difference [42], prediction time lag and the J index [43], and different methods [44-47] based on using error grids developed for blood glucose meter evaluation, such as the Clarke Error Grid [48] and the Parkes (Consensus) Error Grid [49,50]. More importantly, even with the same metric, glycemic prediction models can exhibit noticeable variation in accuracy when applied to different sets of data owing to the nature of data (in silico or in vivo), the amount of data available for each patient, physiological differences between patients, behavioral changes for each patient, and data quality issues. This variance can be partially reduced by using larger data sets, but for many researchers, only limited data are available owing to the fact that blood glucose readings, similar to all medical data, are usually not shared freely because of patient privacy concerns. Although there have been recent attempts to facilitate blood glucose research by creating established sets of CGM data available to scientists, such as the Ohio T1DM (type 1 diabetes mellitus) data set [51], most studies published to date use private data sets for evaluation, which makes it difficult to objectively evaluate the quality of their results.

Furthermore, the prediction accuracy of different studies may be significantly affected by varied availability of non-CGM data, particularly information related to meal and insulin events. If predictions are only made for periods when no such events occur (which can only be done if the researcher has the data indicating their occurrence), or if these events are taken into account by the predictive model, the accuracy is likely to be much higher than in case of making a prediction for an interval during which unknown events affecting the patient’s blood glucose may have taken place.

### Feedback Delays and Implications for Predictions

It is important to point out that CGM devices do not measure the actual blood glucose levels but measure the concentration of glucose in interstitial fluid, which tends to follow blood glucose with a patient- and condition-dependent time lag, usually in the range of 5 to 20 min [52-55]. Although the postprocessing of measured CGM data may partially account for this delay, to avoid out-of-range blood glucose excursions, the predictions need to be made in advance in order for the user (or an automatically controlled insulin pump if the predicted values are used by an artificial pancreas algorithm) to be able to make a correction, while the true blood glucose concentration is still within its reference range.

There are several other sources of delays when using predictions for blood glucose control. Frequently, predictions themselves may be lagging compared with the future interstitial glucose levels because of the nature of the predictive algorithm. Next, CGM devices only perform measurements using discrete time intervals (usually between 3 and 15 min, with 5 min being the most common in practice). Therefore, the last measured point may not be quite up to date at the moment the user sees the prediction. Additional delays are introduced by the CGM filtering algorithms [53]. In addition, the corrective action by the user may not have an immediate effect on blood glucose (eg, even for rapid-acting insulin delivered subcutaneously, the action is delayed by about 5-10 min [56]).

Owing to all these delays, in order for the predictions to be maximally effective in preventing out-of-range blood glucose excursions, it is preferable to anticipate glycemic changes for at least 30 min in advance, especially in cases of hyperglycemic events caused by the delayed action of insulin. For hypoglycemia prediction, shorter time horizons may be acceptable [23], although a longer accurate prediction would still give the user more time to take preventive measures.

### Goals

The aim of this paper is to describe how the challenges that exist in blood glucose predictions are addressed in the Diabits smartphone app and to evaluate the accuracy of its predictions and the potential clinical effects of the app using data from the app’s users and other existing data sets.

## Methods

### General Description of Diabits

Diabits is a smartphone app that is available both for iOS and Android phones, which reads current blood glucose data either from the app associated with a Dexcom CGM device (via Dexcom Share) or from Nightscout, a cloud-based data aggregator project that can collect, if configured by the user, current data from a Dexcom or Medtronic CGM, and then presents these data in real time to the user, along with predictions of blood glucose behavior for the next 60 min and statistical information and charts based on the patient’s past blood glucose data.

**Figure 1:**
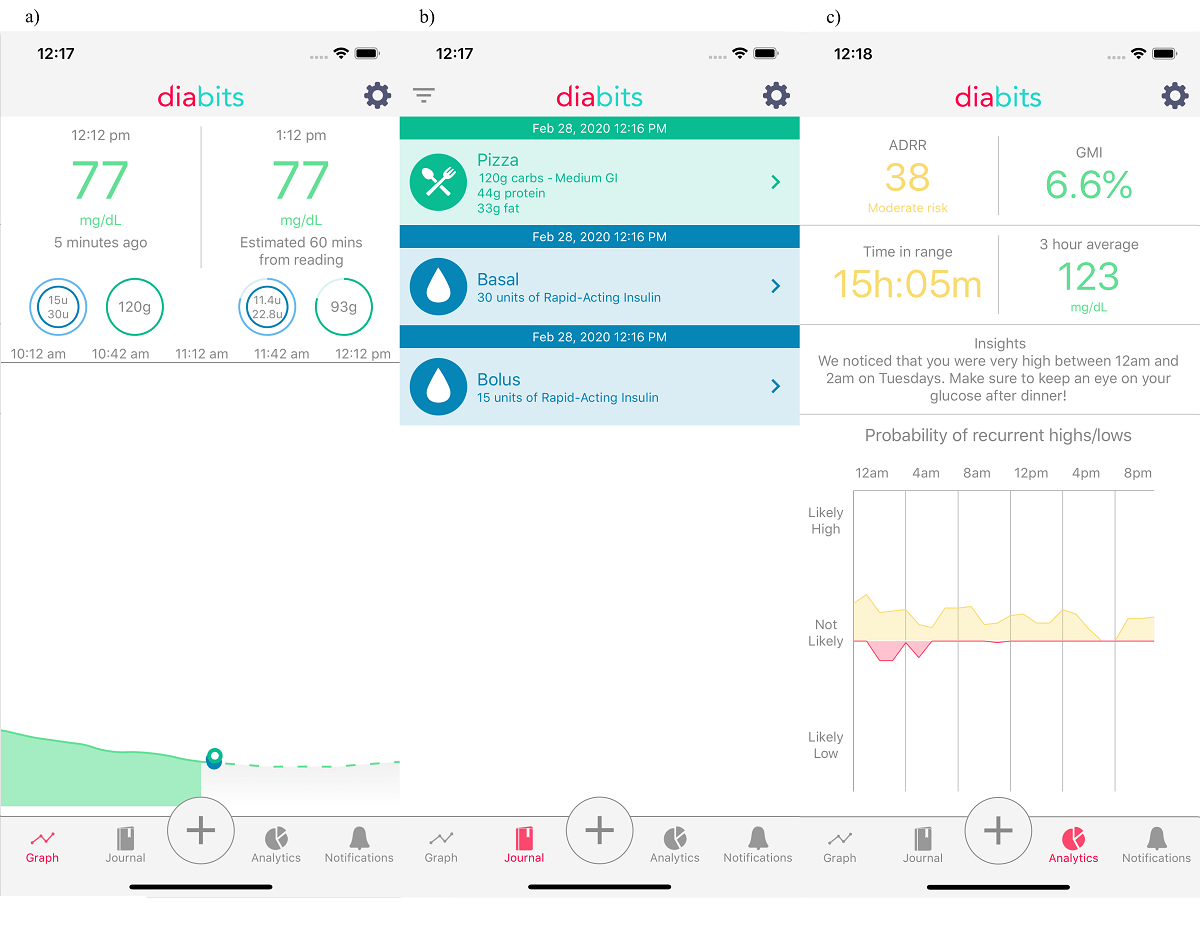
The interface of Diabits, including (a) the Graph panel, showing recent CGM values and the predictions for the next hour; (b) the Journal panel, where the users can enter relevant event information (food, insulin, etc), and see the past history of CGM data and events; (c) the Analytics panel, showing various glycemic statistics and insights that may help the users control their blood glucose levels. CGM: continuous glucose monitoring.

The main parts of the user interface of the app are shown in Figure 1. Graph panel (a) is the main screen of the app, displaying the recent CGM data, predicted future blood glucose values, and estimated values of insulin and carbohydrates *on board*, that is, available for future use by the body. The meal and insulin information, entered manually by each user of the app based on their best knowledge, is displayed in the Journal panel (b). The Analytics panel (c) shows several statistics based on the recent history of the patient’s blood glucose. Some of the graphic parts of the design may have experienced minor changes throughout the study.

The predictive models of Diabits were originally created on the basis of the results of a clinical study conducted in collaboration with the endocrinology unit of BC Children’s Hospital (located in Vancouver, Canada) between April and October 2017 [57]. During this study, CGM data and heart rate and physical activity information of 9 young patients with type 1 diabetes were collected over a period of 2 months with the goal of creating an accurate model for short-term blood glucose predictions. The predictive models that were developed during this study were subsequently refined [58] using data from a larger pool (approximately 1200 people) of free-living users of the app with approximately 1.6 million data points.

**Figure 2:**
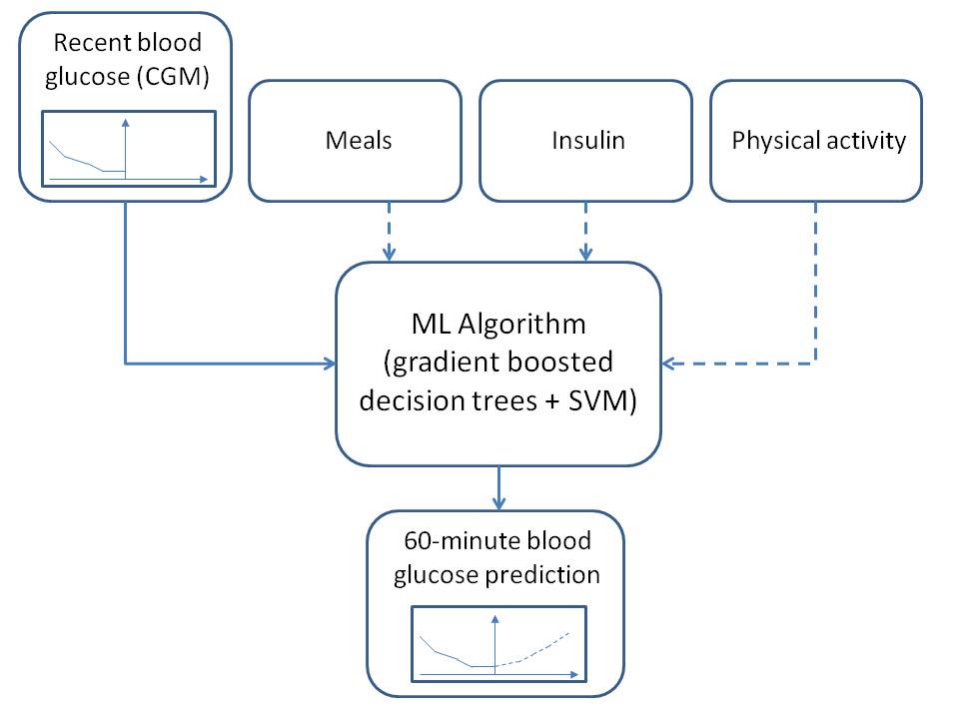
A block diagram of the Diabits predictive module. Solid connections between blocks indicate mandatory data, whereas dashed connections show optional inputs. The dashed line in the bottom block shows the 60-min prediction that was generated by the machine learning algorithm. CGM: continuous glucose monitoring; ML: machine learning; SVM: support vector machine.

The app gives users an option to manually record, according to their knowledge, food consumption (carbohydrate, protein, and fat content and the glycemic index), insulin intake (the number of units and the type of insulin), physical exercise (intensity and duration), and other events that may affect their blood glucose. This information is added to the CGM data as model inputs to increase the prediction accuracy. The predictive models of Diabits rely significantly on CGM inputs, as most users do not provide enough food and insulin information required to make a model that is primarily based on physiological principles. However, all available physiological inputs are taken into account when making a prediction. A schematic diagram of the Diabits prediction approach is shown in Figure 2.

### Details of Machine Learning Approach Used in Diabits

Glucose predictions are made via a supervised machine learning framework, with personalized models trained using each patient’s past data.

Glucose values are calculated for 4 time points: 15, 30, 45, and 60 min ahead, with a separate model trained for each point. When plotting the data for users, the in-between points are filled using cubic interpolation. Although it is possible to train models for any number of minutes divisible by the CGM time step (eg, for 5, 10, 15 min, if the CGM time step is 5 min), it is not necessary in practice because the actual blood glucose behavior of patients with insulin-dependent diabetes typically lacks a noticeable high-frequency component [59] (even though unfiltered CGM values may exhibit such fluctuations because of random measurement errors).

To create inputs for the model, in addition to CGM data, recent food and insulin records, if available, are used to estimate the amount of carbohydrates and insulin currently present in the body (this information is also displayed for the user to see) and their rates of utilization. The calculations are performed using physiological models similar to those reported in the literature, (eg, [12,60]). As these physiological models have a number of parameters that are specific to each patient, these calculations can only be performed once a sufficient number of previous points with food and insulin data have been collected so that personalized parameters can be estimated from these. Until that point (for newer app users and those who rarely provide such data to the app), a simpler estimation approach for the current amount of carbohydrates and insulin remaining is used based on the food and insulin information reported by the patient, each patient’s insulin-to-carbohydrate ratio and correction factor provided to the app at sign-up, and the changes in blood glucose levels since each food and/or insulin event.

Other data points, such as those related to the time of the day, day of the week, and recent physical activity data, are also added as separate model inputs to increase the accuracy of predictions.

The resulting inputs are used for training a model that combines gradient boosted decision trees and SVM regression. Gradient boosted decision trees [61] is an ensemble machine learning technique that works by consecutively training new trees on the differences between the ground truth labels and the combined prediction of all preceding trees. SVM regression [62] operates similar to linear regression, but with a maximum margin (hinge) loss and a kernel mapping that allows to model nonlinear systems. Diabits uses standard implementations of both of these algorithms from open-source Python packages.

The exact mechanism by which these two methods are implemented and combined are not addressed in this paper but may be disclosed in future publications. Generally, the decision tree model is used to evaluate which of the several possible physiological states the patient is currently in, and then an SVM model trained exclusively on the data pertaining to this particular state (as determined by the training algorithm) generates the prediction.

For each Diabits user, the initial personalized (based solely on this user’s data) model is built once 2000 CGM points (about a week of continuous data) are available. Thereafter, the model is retrained every 2 weeks to take advantage of the most recent data.

### Prediction Adjustments in Diabits

One of the issues that needs to be addressed when predictive models are trained on past patient behavior is that in the absence of detailed nutritional and insulin information for free-living patients, training points may reflect unrecorded prior corrections that the patients have made by either ingesting carbohydrates or using insulin. This is particularly problematic when blood glucose is near the edges of the target range (eg, just above 70 mg/dL or just below 180 mg/dL for the standard reference range of glucose values). A model trained on such data will likely predict similar corrections happening in the future, which may result in the patient actually foregoing necessary corrections owing to the fact that blood glucose is predicted to normalize on its own.

To mitigate this effect, in situations where such errors are likely to occur (ie, in situations with an impending hypo- or hyperglycemic event that the user is likely to have avoided in the past training data by taking food or insulin), Diabits uses an additional algorithm to correct its predictions to generate the most likely trajectory of blood glucose in the absence of future external interventions. The user can then decide, based on their own judgment, if any interventions are necessary. This adjustment is only used when blood glucose is trending toward the outside of the target range, there has been no recent change in the direction of the trend indicating a possible unreported meal or insulin event, and no meal or insulin events have been reported in the last 40 min. The final prediction is generated as a weighted average of the main model’s prediction and a prediction that applies linear regression to the recent CGM data and therefore is guaranteed to continue the current trend.

Note that this Diabits adjustment, which typically increases the calculated prediction error (because we are no longer trying to predict what will actually happen, but instead what will happen if no action is taken) but, in our opinion, makes the predictions more practically useful, was not used to ensure a fair comparison in part III of the results of this paper, namely when comparing the prediction accuracy of our model with published research on the Ohio T1DM data set. The results for the actual in-app predictions and glycemic control versus frequency of app use (part I and part II), however, are based on a model that does include this adjustment.

### Study Format and Ethical Compliance

All parts of this research are based on retrospective observational cohort studies. The first part (*Accuracy of Past In-App Predictions for Free-Living Users*) and the second part (*Glycemic Control vs Frequency of App Use*) analyzed the past data of free-living Diabits users. The researchers, in accordance with the Diabits’ privacy policy, had no access to personally identifiable information of the users, relying instead on anonymized randomly generated universally unique identifier strings [63], and had no contact with any of the participants. Thus, we believe that the participants did not fall under the definition of human subjects [64]; hence, no institutional review board review was necessary. Informed consent was received from every Diabits user upon sign-up that their anonymized data could be used for research purposes.

In the third part of the study (*Accuracy of Predictions on the 2018 Ohio T1DM Data Set*), a publicly available anonymized 2018 Ohio T1DM data set [51] was used. The data user agreement for this data set allows the use of its data for research purposes.

#### Part I: Accuracy of Past In-App Predictions for Free-Living Users

The goal of this part of the study was to examine a large set of past Diabits predictions made for the actual users of the app and to determine the clinical safety of these predictions using Clarke and Parkes Error Grid analysis. All of Diabits users with type 1 diabetes (as reported by the patients themselves during sign-up) were ranked by the number of blood glucose data points they shared with the app in 2019, and the 500 patients with the most points were chosen for analysis. The sex and age of each specific subject was not known to the researchers; however, in general, there are many Diabits users in all age categories, from newborn to those older than 70 years, and of different sexes (approximately evenly split between males and females). All of the CGM devices used by the study participants were among those compatible with the app (*General Description of Diabits*). The investigators did not have any further information regarding specific device models for each participant.

The distribution between the Clarke and Parkes Error Grid zones of actual 15-, 30-, 45-, and 60-min predictions made by the app in real time, as compared with the ground-truth data from future CGM points, was calculated using all of the points for these 500 patients where the prediction was made and all of the ground-truth labels were available (6,864,130 total points). The results were examined to determine whether the predictions provided could potentially have led to adverse patient outcomes.

#### Part II: Glycemic Control Versus Frequency of App Use

The goal of this part of the study was to determine whether there is a correlation between how often the users look at the blood glucose graph of Diabits during each day and their blood glucose control. A total of 280 Diabits users who had at least 180 days of CGM data recorded by the app in 2018 to 2019 were included. The patients came from the same pool as in the first part of the study (in fact, many are the same patients); however, their data from 2 calendar years (2018 and 2019) were used for analysis.

The blood glucose control metrics that were calculated included the average blood glucose and its SD, time in euglycemic range (TIR) [65], glucose management indicator (GMI) [66], and high BGI (HBGI) and low BGI (LBGI) blood glucose risk indices [67].

All of the metrics were analyzed as functions of the frequency of daily use, which was defined as the number of times a Diabits user looked at the graph containing CGM values and future blood glucose predictions during 1 calendar day. Diabits records each user’s CGM data as long as the app is running on the smartphone even if the user is not actively looking at the results, so days with zero sessions were included.

The hypothesis of the study was that all of the blood glucose control metrics would improve with more frequent use of the app. All of the users’ days were categorized into 4 different groups, namely those with 0 sessions, 1 to 5 sessions, 6 to 10 sessions, and more than 10 sessions. *P* values, calculated using a one-sided *t* test, are reported for the difference of each metric from that in the group with zero daily sessions (no active use of the app; *P*_0_) and in the closest group with fewer sessions (*P*_fewer_). A value α=.01 was used for the alpha level of significance in all cases, using the Bonferroni correction [68] for multiple comparisons.

#### Part III: Accuracy of Predictions on the 2018 Ohio T1DM Data Set

To facilitate the comparison of the predictive accuracy of Diabits with existing research, the base Diabits prediction framework was applied without any data set–specific adjustments to the data from the Ohio T1DM data set [51] that was used in 2018 Blood Glucose Level Prediction (BGLP) challenge at the third International Workshop on Knowledge Discovery in Healthcare Data.

Using the training portion of the data in the 2018 Ohio T1DM data set, personalized Diabits models were created for each of the 6 patients in the data set. Next, 30-min predictions were generated for all points in the test portion of the data except for the first hour, and the prediction error (RMSE) was calculated and compared against the published results of the challenge [18,30-33,35,40,41].

The CGM data were used as is (no averaging or smoothing to eliminate random errors), and only past and present data (CGM glucose levels, basal and bolus insulin, meal, and exercise information) were used for each point to make predictions. In other words, the data were used in the same manner it is normally used in Diabits, with the training data used to train each patient’s personalized prediction models and the test data to generate predictions and calculate their accuracy.

## Results

### Part I: Accuracy of Past In-App Predictions for Free-Living Users

**Figure 3:**
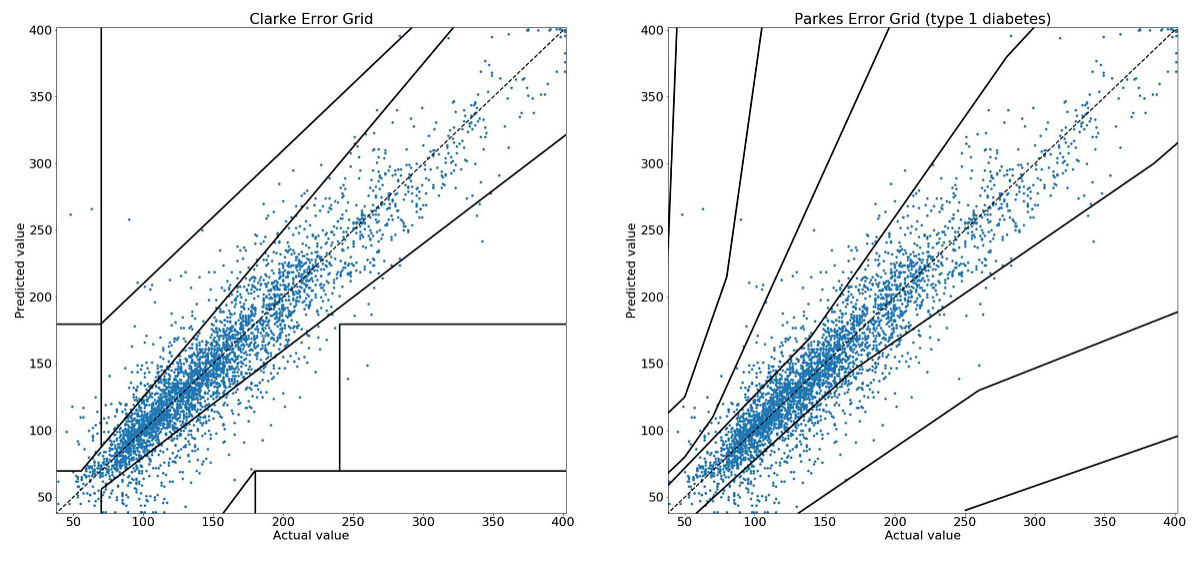
A sample scatter graph of blood glucose values predicted 30 min in advance by the Diabits model versus measured CGM values, plotted against Clarke (left) and Parkes (right) Error Grids.

Actual 30-min Diabits predictions under free-living conditions for the 500 most active patients in 2019 (approximately 6.8 million points) made using personalized models based on the gradient boosted decision trees and the SVM regression algorithm discussed above and evaluated using Parkes Error Grid were found to be 86.89% (5,963,930/6,864,130) clinically accurate (zone A) and 99.56% (6,833,625/6,864,130) clinically acceptable (zones A and B). For the 60-min predictions, the results were 70.56% (4,843,605/6,864,130) clinically accurate and 97.49% (6,692,165/6,864,130) clinically acceptable (Table 1). A sample distribution of predicted values plotted against actual values for both Clarke and Parkes Error Grids is shown in Figure 3.

**Table 1:** Results of error grid analysis of the prediction accuracy of Diabits based on 6,864,130 actual predictions made for 500 most active app users in 2019.

Minutes and error grid type		A, n (%)^a^	B, n (%)^a^	C, n (%)^a^	D, n (%)^a^	E, n (%)^a^
**15**						
	Clarke	6,565,030 (95.64)	278,954 (4.07)	135 (0.00)	19,981 (0.29)	30 (0.00)
	Parkes	6,613,321 (96.34)	246,767 (3.60)	3968 (0.06)	71 (0.00)	3 (0.00)
**30**						
	Clarke	5,835,511 (85.01)	964,979 (14.06)	3276 (0.05)	59,834 (0.87)	530 (0.01)
	Parkes	5,963,930 (86.89)	869,695 (12.67)	29,587 (0.43)	915 (0.01)	3 (0.00)
**45**						
	Clarke	5,174,795 (75.39)	1,559,461 (22.72)	21,510 (0.31)	103,629 (1.51)	4735 (0.07)
	Parkes	5,359,782 (78.08)	1,414,438 (20.61)	85,974 (1.25)	3931 (0.06)	5 (0.00)
**60**						
	Clarke	4,626,623 (67.40)	2,024,709 (29.50)	55,195 (0.80)	144,512 (2.11)	13,091 (0.19)
	Parkes	4,843,605 (70.56)	1,848,560 (26.93)	162,537 (2.37)	9416 (0.14)	12 (0.00)

^a^The numbers show the percentage of prediction points in each zone of the Clarke and the Parkes Error Grid. For both grids, the zones are defined as clinically accurate (A), clinically acceptable (B), and clinically inaccurate (C-E) [48-50].

### Part II: Glycemic Control Versus Frequency of App Use

To evaluate the correlation between the daily frequency of Diabits use and the quality of blood glucose control, several commonly used blood glucose control metrics were calculated for 280 users who had at least 180 days of CGM data recorded by the app in 2018 to 2019 (86,973 days combined for all users) as a function of daily number of sessions (ie, the times the user opened the app to look at the blood glucose graph) with Diabits (Table 2).

As can be seen from Table 2, all of the metrics except LBGI were better for days with more frequent Diabits use (in almost all cases, P/2<α/36=.00027, the latter value being the significance level calculated using the Bonferroni correction formula for multiple comparisons, thus indicating a statistically significant positive correlation). In the case of LBGI, there was a very slight statistically significant increase in hypoglycemic risk when using the app more frequently (as could be expected owing to tighter glucose control); however, all of the values were well within the minimal risk region of LBGI<1.1 [69].

**Table 2:** Various metrics of blood glucose control as a function of frequency of daily Diabits use.

Daily sessions^a^	0	1-5	6-10	>10
Average blood glucose (mg/dL)	154.0	150.7; *P*_0_<.001;^b^ *P*_fewer_<.001	145.6; *P*_0_<.001; *P*_fewer_<.001	141.6; *P*_0_<.001; *P*_fewer_<.001
Standard deviation (mg/dL)	47.6	45.3; *P*_0_<.001; *P*_fewer_<.001	42.1; *P*_0_<.001; *P*_fewer_<.001	41.5; *P*_0_<.001; *P*_fewer_=.07
Time in euglycemic range, as % of all data	67.52	69.39%; *P*_0_<.001; *P*_fewer_<.001	73.05%; *P*_0_<.001; *P*_fewer_<.001	74.28%; *P*_0_<.001; *P*_fewer_=.004
GMI^c^ (%)	6.99	6.91%; *P*_0_<.001; *P*_fewer_<.001	6.79%; *P*_0_<.001; *P*_fewer_<.001	6.70%; *P*_0_<.001; *P*_fewer_<.001
HBGI^d^ (<4.5: low risk; 4.5-9.0: moderate risk; >9.0: high risk) [67]	4.63	4.20; *P*_0_<.001; *P*_fewer_<.001	3.62; *P*_0_<.001; *P*_fewer_<.001	3.13; *P*_0_<.001; *P*_fewer_<.001
LBGI^e^ (<1.1: minimal risk; 1.1-2.5: low risk; 2.5-5.0: moderate risk; >5.0: high risk) [69]	0.42	0.45; *P*_0_<.001; *P*_fewer_<.001	0.46; *P*_0_=.007; *P*_fewer_=.32	0.59; *P*_0_<.001; *P*_fewer_<.001

^a^Daily sessions refers to the number of times a Diabits user looks at the CGM values and predictions during 1 calendar day. Diabits records each user’s CGM data as long as the application is running on the smartphone even if the user is not actively looking at the results, so days with 0 sessions are included.

^b^All *P* values <.001 are reported as *P*<.001. *P*_0_ and *P*_fewer_ are defined in the methods section of this paper.

^c^GMI: glucose management indicator.

^d^HBGI: high blood glucose risk index.

^e^LBGI: low blood glucose risk index.

### Part III: Accuracy of Predictions on the 2018 Ohio T1DM Data Set

The calculated RMSE values for Diabits predictions on the test portion of the 2018 Ohio T1DM data set [51] are presented in Table 3.

Of note, the mean prediction error of the Diabits base model (18.68 mg/dL) is lower than that of all other published results.

**Table 3:** Root mean square error (mg/dL) of 30-min prediction accuracy of the base Diabits model for 6 patients in the 2018 Ohio type 1 diabetes mellitus data set compared with the best of the published results of 2018 Blood Glucose Level Prediction Challenge [51] on the same data.

Predictive model (RMSE^a^, mg/dL)	Patient number						Mean (SD)^b^	
	559	563	570	575	588	591		
Diabits base model	*17.94* ^c^	18.29	*15.44*	22.22	17.53	20.64		*18.68* (2.19)
Martinsson, 2019 (LSTM RNN^d^) [35]	18.77	*17.96*	15.96	*21.68*	18.54	*20.29*		18.87 (1.79)
Chen, 2018 (DRNN^e^) [31]	18.78	18.12	15.46	22.83	17.73	21.34		19.04 (2.42)
Bertachi, 2018 (feed-forward NN^f^) [33]	18.83	19.43	15.88	22.86	17.84	21.12		19.33 (2.24)
Xie, 2018 (SVM^g^) [18]	18.19	19.12	15.67	24.61	*17.49*	22.12		19.53 (2.99)
Xie, 2018 (ARX linear regression) [18]	18.36	19.02	16.03	23.90	18.25	21.99		19.59 (2.60)
Martinsson, 2018 (LSTM RNN) [30]	19.50	19.00	16.50	24.20	19.20	22.00		20.07 (2.44)
Midroni, 2018 (XGBoost) [40]	19.81	18.42	18.14	24.17	19.24	22.49		20.38 (2.21)
Contreras, 2018 (Grammatical evolution) [41]	20.98	19.36	19.55	24.49	20.45	22.28		21.19 (1.77)
Zhu, 2018 (WaveNet convolutional NN) [32]	21.72	20.17	18.03	24.80	21.42	24.22		21.73 (2.30)

^a^RMSE: root mean square error.

^b^The mean column is calculated by averaging the 6 previous columns (mean root mean square error over all patients).

^c^The best result for each patient is highlighted in italics.

^d^RNN: recurrent neural network.

^e^DRNN: dilated recurrent neural network.

^f^NN: neural network.

^g^SVM: support vector machine.

## Discussion

### Principal Findings

This paper has studied the predictive accuracy of Diabits, a smartphone app that performs blood glucose monitoring based on CGM data, presents a statistical analysis of past data, and generates short-term (up to 60 min) predictions of future glucose behavior. In addition, the correlation between daily use of Diabits and blood glucose control metrics of its users was examined.

A large number of actual predictions made by Diabits for its users were evaluated using the Clarke and Parkes Error Grid, and the resulting values were found to be in the clinically acceptable range 97.49% of the time (6,692,165/6,864,130) for 60-min predictions and 99.56% of the time (6,833,625/6,864,130) for 30-min predictions on the Parkes Grid (with similar results for the Clarke Grid), which showed that the vast majority of predictions were accurate enough to not adversely affect the patients.

By analyzing the results of actual app use, it was statistically established that more frequent daily use of Diabits was correlated with improvement in many blood glucose control metrics, including average blood glucose and its SD, TIR, GMI, and HBGI. This is consistent with the goal of the app to help patients better manage their blood glucose and pre-emptively avoid hyper- or hypoglycemia.

Finally, the accuracy of Diabits was directly compared with that of existing research using predictions on the 2018 Ohio T1DM data set, with the resulting RMSE being lower than that in the studies published by other researchers [18,30-33,35,40,41].

All of these results show the viability of Diabits as an effective tool for blood glucose control in CGM users. They also support the quality of the model underlying Diabits to make informative blood glucose predictions based on personalized machine learning models.

### Strengths, Limitations, and Possible Future Developments

In part I, the accuracy of the actual glycemic predictions of Diabits was calculated using more than 6.8 million data points. This provided a solid statistical basis for the calculations and ensured the validity of the results.

The combination of gradient boosting decision trees and SVM regression in the Diabits models may have provided an additional ensembling [70] benefit that enhanced the prediction accuracy. In addition, we believe that one of the reasons why Diabits personalized models based on these techniques work particularly well for most patients compared with, for example, neural network models, is the somewhat limited amount of training data available for each patient, which favors the traditional machine learning techniques. However, the downside is that the current personalized approach fails to take advantage of the global pool of data available through the app. One possible future research direction is to use combined data from a large number of patients to train a deep neural network model (which may achieve better accuracy with a large amount of data), and then fine-tune this model for each patient.

In part II, the discovered correlation between the daily use of Diabits and the improvement in blood glucose control metrics was based on more than 86,000 days of app use, once again giving the results statistical significance. However, the observational nature of the study and the lack of knowledge of which, if any, corrections were made by the users based on the app output does not allow us to establish causality or estimate the level of importance of each feature of Diabits, which may be a topic of future research.

In part III, the predictions of Diabits on the 2018 Ohio T1DM data set showed an improved average RMSE for 30-min predictions over other published approaches, demonstrating Diabits’ high predictive accuracy when compared with other leading models on the same data set.

## Abbreviations

BCT: Bio Conscious Technologies, Inc
BGLP: Blood Glucose Level Prediction
CGM: continuous glucose monitoring
GMI: glucose management indicator
HBGI: high blood glucose risk index
LBGI: low blood glucose risk index
RMSE: root mean square error
SVM: support vector machine
T1DM: type 1 diabetes mellitus
TIR: time in euglycemic range

## References

[1] World Health Organization (2016). Global Report on Diabetes.

[2] Klein R (1995). Hyperglycemia and microvascular and macrovascular disease in diabetes. Diabetes Care.

[3] Leahy J, Clark N, Cefalu W (2000). Medical Management of Diabetes Mellitus.

[4] Workgroup on Hypoglycemia‚ American Diabetes Association (2005). Defining and reporting hypoglycemia in diabetes: a report from the American Diabetes Association Workgroup on Hypoglycemia. Diabetes Care.

[5] Rodbard D (2016). Continuous glucose monitoring: a review of successes, challenges, and opportunities. Diabetes Technol Ther.

[6] Cobelli C, Renard E, Kovatchev B (2011). Artificial pancreas: past, present, future. Diabetes.

[7] Bequette BW (2012). Challenges and recent progress in the development of a closed-loop artificial pancreas. Annu Rev Control.

[8] Bergman R, Phillips L, Cobelli C (1981). Physiologic evaluation of factors controlling glucose tolerance in man: measurement of insulin sensitivity and beta-cell glucose sensitivity from the response to intravenous glucose. J Clin Invest.

[9] Sorensen J A Physiologic Model of Glucose Metabolism in Man and Its Use to Design and Assess Improved Insulin Therapies for Diabetes. Carnegie Mellon School of Computer Science.

[10] Caumo A, Simeoni M, Cobelli C (2001). Glucose modelling. Modelling Methodology for Physiology and Medicine.

[11] Makroglou A, Li J, Kuang Y (2006). Mathematical models and software tools for the glucose-insulin regulatory system and diabetes: an overview. Appl Numer Math.

[12] Dalla Man C, Rizza RA, Cobelli C (2007). Meal simulation model of the glucose-insulin system. IEEE Trans Biomed Eng.

[13] Georga E, Protopappas V, Fotiadis D (2011). Glucose prediction in type 1 and type 2 diabetic patients using data-driven techniques. Knowledge-Oriented Applications in Data Mining.

[14] Sparacino G, Zanderigo F, Corazza S, Maran A, Facchinetti A, Cobelli C (2007). Glucose concentration can be predicted ahead in time from continuous glucose monitoring sensor time-series. IEEE Trans Biomed Eng.

[15] Reifman J, Rajaraman S, Gribok A, Ward WK (2007). Predictive monitoring for improved management of glucose levels. J Diabetes Sci Technol.

[16] Eren-Oruklu M, Cinar A, Quinn L, Smith D (2009). Estimation of future glucose concentrations with subject-specific recursive linear models. Diabetes Technol Ther.

[17] Lu Y, Rajaraman S, Ward W (2011). Predicting Human Subcutaneous Glucose Concentration in Real Time: a Universal Data-Driven Approach. Annual International Conference of the IEEE Engineering in Medicine and Biology Society.

[18] Xie J, Wang Q (2018). Benchmark machine learning approaches with classical time series approaches on the blood glucose level prediction challenge. http://ceur-ws.org/Vol-2148/paper16.pdf.

[19] Bequette B (2004). Optimal Estimation Applications to Continuous Glucose Monitoring. Proceedings of the 2004 American Control Conference.

[20] Eberle C, Ament C (2011). The Unscented Kalman Filter estimates the plasma insulin from glucose measurement. Biosystems.

[21] Wang Q, Molenaar P, Harsh S, Freeman K, Xie J, Gold C, Rovine M, Ulbrecht J (2014). Personalized State-space Modeling of Glucose Dynamics for Type 1 Diabetes Using Continuously Monitored Glucose, Insulin Dose, and Meal Intake: An Extended Kalman Filter ApproachPersonalized state-space modeling of glucose dynamics for type 1 diabetes using continuously monitored glucose, insulin dose, and meal intake: an extended kalman filter approach. J Diabetes Sci Technol.

[22] Toffanin C, del Favero S, Aiello E, Messori M, Cobelli C, Magni L (2018). Glucose-insulin model identified in free-living conditions for hypoglycaemia prevention. J Process Control.

[23] Toffanin C, Aiello EM, Cobelli C, Magni L (2019). Hypoglycemia prevention via personalized glucose-insulin models identified in free-living conditions. J Diabetes Sci Technol.

[24] Mougiakakou S, Prountzou A, Iliopoulou D (2006). Neural Network-based Glucose-Insulin Metabolism Models for Children With Type 1 Diabetes. International Conference of the IEEE Engineering in Medicine and Biology Society.

[25] Pappada SM, Cameron BD, Rosman PM (2008). Development of a neural network for prediction of glucose concentration in type 1 diabetes patients. J Diabetes Sci Technol.

[26] Pérez-Gandía C, Facchinetti A, Sparacino G, Cobelli C, Gómez EJ, Rigla M, de Leiva A, Hernando M (2010). Artificial neural network algorithm for online glucose prediction from continuous glucose monitoring. Diabetes Technol Ther.

[27] Zecchin C, Facchinetti A, Sparacino G, De Nicolao G, Cobelli C (2012). Neural network incorporating meal information improves accuracy of short-time prediction of glucose concentration. IEEE Trans Biomed Eng.

[28] Mhaskar H, Pereverzyev S, van der Walt MD (2017). A deep learning approach to diabetic blood glucose prediction. Front Appl Math Stat.

[29] Mirshekarian S, Bunescu R, Marling C (2017). Using LSTMs to Learn Physiological Models of Blood Glucose Behavior. 39th Annual International Conference of the IEEE Engineering in Medicine and Biology Society.

[30] Martinsson J, Schliep A, Eliasson B (2018). Automatic Blood Glucose Prediction With Confidence Using Recurrent Neural Networks. http://ceur-ws.org/Vol-2148/paper10.pdf.

[31] Chen J, Li K, Herrero P (2018). Dilated recurrent neural network for short-time prediction of glucose concentration. http://ceur-ws.org/Vol-2148/paper11.pdf.

[32] Zhu T, Li K, Herrero P (2018). A deep learning algorithm for personalized blood glucose prediction. http://ceur-ws.org/Vol-2148/paper12.pdf.

[33] Bertachi A, Biagi L, Contreras I (2018). Prediction of Blood Glucose Levels and Nocturnal Hypoglycemia Using Physiological Models and Artificial Neural Networks. http://ceur-ws.org/Vol-2148/paper14.pdf.

[34] Li K, Daniels J, Liu C, Herrero P, Georgiou P (2020). Convolutional Recurrent Neural Networks for Glucose Prediction. IEEE J Biomed Health Inform.

[35] Martinsson J, Schliep A, Eliasson B, Mogren O (2019). Blood glucose prediction with variance estimation using recurrent neural networks. J Healthc Inform Res.

[36] Aiello E, Lisanti G, Magni L, Musci M, Toffanin C (2020). Therapy-driven deep glucose forecasting. Eng Appl Artif Intell.

[37] Georga E, Protopappas V, Ardigo D, Marina M, Zavaroni I, Polyzos D, Fotiadis Di (2013). Multivariate prediction of subcutaneous glucose concentration in type 1 diabetes patients based on support vector regression. IEEE J Biomed Health Inform.

[38] Plis K, Bunescu R, Marling C (2014). A machine learning approach to predicting blood glucose levels for diabetes management. https://www.aaai.org/ocs/index.php/WS/AAAIW14/paper/download/8737/8308%20.

[39] Georga E, Protopappas V, Polyzos D, Fotiadis DI (2012). A predictive model of subcutaneous glucose concentration in type 1 diabetes based on Random Forests. Conf Proc IEEE Eng Med Biol Soc.

[40] Midroni C, Leimbigler P, Baruah G (2020). Predicting glycemia in type 1 diabetes patients: experiments with XGBoost. http://ceur-ws.org/Vol-2148/paper13.pdf.

[41] Contreras I, Bertachi A, Biagi L (2020). Using grammatical evolution to generate short-term blood glucose prediction models. http://ceur-ws.org/Vol-2148/paper15.pdf.

[42] Reiterer F, Polterauer P, Schoemaker M, Schmelzeisen-Redecker G, Freckmann G, Heinemann L, del Re L (2017). Significance and reliability of MARD for the accuracy of CGM systems. J Diabetes Sci Technol.

[43] Facchinetti A, Sparacino G, Trifoglio E, Cobelli C (2011). A new index to optimally design and compare continuous glucose monitoring glucose prediction algorithms. Diabetes Technol Ther.

[44] Kovatchev BP, Gonder-Frederick LA, Cox DJ, Clarke WL (2004). Evaluating the accuracy of continuous glucose-monitoring sensors: continuous glucose-error grid analysis illustrated by TheraSense freestyle navigator data. Diabetes Care.

[45] Wentholt IM, Hoekstra JB, Devries JH (2006). A critical appraisal of the continuous glucose-error grid analysis. Diabetes Care.

[46] Clarke W, Anderson S, Kovatchev B (2008). Evaluating clinical accuracy of continuous glucose monitoring systems: continuous glucose-error grid analysis (CG-EGA). Curr Diabetes Rev.

[47] Sivananthan S, Naumova V, Man CD, Facchinetti A, Renard E, Cobelli C, Pereverzyev SV (2011). Assessment of blood glucose predictors: the prediction-error grid analysis. Diabetes Technol Ther.

[48] Clarke WL, Cox D, Gonder-Frederick LA, Carter W, Pohl SL (1987). Evaluating clinical accuracy of systems for self-monitoring of blood glucose. Diabetes Care.

[49] Parkes JL, Slatin SL, Pardo S, Ginsberg BH (2000). A new consensus error grid to evaluate the clinical significance of inaccuracies in the measurement of blood glucose. Diabetes Care.

[50] Pfützner A, Klonoff DC, Pardo S, Parkes JL (2013). Technical aspects of the Parkes error grid. J Diabetes Sci Technol.

[51] Marling C, Bunescu R (2018). The OhioT1DM Dataset for Blood Glucose Level Prediction. http://ceur-ws.org/Vol-2148/paper09.pdf.

[52] Keenan DB, Mastrototaro JJ, Voskanyan G, Steil GM (2009). Delays in minimally invasive continuous glucose monitoring devices: a review of current technology. J Diabetes Sci Technol.

[53] Rebrin K, Sheppard NF, Steil GM (2010). Use of subcutaneous interstitial fluid glucose to estimate blood glucose: revisiting delay and sensor offset. J Diabetes Sci Technol.

[54] Basu A, Dube S, Veettil S, Slama M, Kudva YC, Peyser T, Carter RE, Cobelli C, Basu R (2015). Time lag of glucose from intravascular to interstitial compartment in type 1 diabetes. J Diabetes Sci Technol.

[55] Cobelli C, Schiavon M, Dalla Man C, Basu A, Basu R (2016). Interstitial fluid glucose is not just a shifted-in-time but a distorted mirror of blood glucose: insight from an in Silico study. Diabetes Technol Ther.

[56] Fath M, Danne T, Biester T, Erichsen L, Kordonouri O, Haahr H (2017). Faster-acting insulin aspart provides faster onset and greater early exposure vs insulin aspart in children and adolescents with type 1 diabetes mellitus. Pediatr Diabetes.

[57] Hayeri A (2018). Predicting future glucose fluctuations using machine learning and wearable sensor data. https://diabetes.diabetesjournals.org/content/67/Supplement_1/738-P.

[58] Hayeri A (2019). Diabits - an AI-powered smartphone application for blood glucose monitoring and predictions. https://diabetes.diabetesjournals.org/content/68/Supplement_1/922-P.

[59] Gough DA, Kreutz-Delgado K, Bremer TM (2003). Frequency characterization of blood glucose dynamics. Ann Biomed Eng.

[60] Man CD, Micheletto F, Lv D, Breton M, Kovatchev B, Cobelli C (2014). The UVA/PADOVA type 1 diabetes simulator: new features. J Diabetes Sci Technol.

[61] Mason L, Baxter J, Bartlett P (1999). Boosting Algorithms as Gradient Descent. https://dl.acm.org/doi/10.5555/3009657.3009730.

[62] Drucker H, Burges C, Kaufman L (1996). Support Vector Regression Machines. https://dl.acm.org/doi/10.5555/2998981.2999003.

[63] Leach P, Mealling M, Salz R A Universally Unique IDentifier (UUID) URN Namespace. Internet Engineering Task Force.

[64] 45 CFR 46. The US Department of Health and Human Services (HHS).

[65] Battelino T, Danne T, Bergenstal RM, Amiel SA, Beck R, Biester T, Bosi E, Buckingham BA, Cefalu WT, Close KL, Cobelli C, Dassau E, DeVries JH, Donaghue KC, Dovc K, Doyle FJ, Garg S, Grunberger G, Heller S, Heinemann L, Hirsch IB, Hovorka R, Jia W, Kordonouri O, Kovatchev B, Kowalski A, Laffel L, Levine B, Mayorov A, Mathieu C, Murphy HR, Nimri R, Nørgaard K, Parkin CG, Renard E, Rodbard D, Saboo B, Schatz D, Stoner K, Urakami T, Weinzimer SA, Phillip M (2019). Clinical targets for continuous glucose monitoring data interpretation: recommendations from the international consensus on time in range. Diabetes Care.

[66] Bergenstal RM, Beck RW, Close KL, Grunberger G, Sacks DB, Kowalski A, Brown AS, Heinemann L, Aleppo G, Ryan DB, Riddlesworth TD, Cefalu WT (2018). Glucose management indicator (GMI): a new term for estimating A1C from continuous glucose monitoring. Diabetes Care.

[67] Kovatchev B, Straume M, Cox D, Farhy Ls (2000). Risk Analysis of Blood Glucose Data: A Quantitative Approach to Optimizing the Control of Insulin Dependent Diabetes. Journal of Theoretical Medicine.

[68] Miller RG (1966). Simultaneous Statistical Inference.

[69] Kovatchev BP, Cox DJ, Kumar A, Gonder-Frederick L, Clarke WL (2003). Algorithmic evaluation of metabolic control and risk of severe hypoglycemia in type 1 and type 2 diabetes using self-monitoring blood glucose data. Diabetes Technol Ther.

[70] Rokach L (2009). Ensemble-based classifiers. Artif Intell Rev.

